# Development of Artificial Synthetic Pathway of Endophenazines in *Pseudomonas chlororaphis* P3

**DOI:** 10.3390/biology11030363

**Published:** 2022-02-24

**Authors:** Ying Liu, Shengjie Yue, Muhammad Bilal, Malik Jan, Wei Wang, Hongbo Hu, Xuehong Zhang

**Affiliations:** 1 State Key Laboratory of Microbial Metabolism, School of Life Sciences and Biotechnology, Shanghai Jiao Tong University, Shanghai 200240, China; liuyinger@sjtu.edu.cn (Y.L.); yuesj_sjtu@163.com (S.Y.); jan.malik@sjtu.edu.cn (M.J.); weiwang100@sjtu.edu.cn (W.W.); hbhu@sjtu.edu.cn (H.H.); 2 School of Life Science and Food Engineering, Huaiyin Institute of Technology, Huaian 223003, China; bilaluaf@hyit.edu.cn; 3 Shanghai Nongle Joint R&D Center on Biopesticides and Biofertilizers, Shanghai Jiao Tong University, Shanghai 200240, China

**Keywords:** endophenazines, prenyltransferase, microbial synthesis, *P. chlororaphis*, metabolic engineering

## Abstract

**Simple Summary:**

Terpenoid phenazines generally produced in *Streptomyces* exhibit potential antitumor and antibacterial activities. In this study, we designed and constructed an artificial biosynthetic pathway for the synthesis of terpenoid phenazines in *Pseudomonas chlororaphis* P3. We successfully synthesized endophenazine A and endophenazine A1 for the first time in *Pseudomonas* by introducing the prenyltransferase PpzP. Moreover, we revealed the biosynthetic pathway of endophenazine A1 in *P. chlororaphis* P3. This study enriches the diversity of phenazines in *P. chlororaphis* P3 and provides a reference for the heterologous synthesis of terpenoid phenazines.

**Abstract:**

Endophenazine A is a terpenoid phenazine with phenazine-1-carboxylic acid (PCA), and dimethylallyl diphosphate (DMAPP) derived from the 2-methyl-D-erythritol-4-phosphate (MEP) pathway as the precursor, which shows good antimicrobial activity against several Gram-positive bacteria and fungi. However, the highest yield of endophenazine A was about 20 mg/L in *Streptomyces*, limiting its large-scale industrial development. *Pseudomonas chlororaphis* P3, possessing an efficient PCA synthesis and MEP pathways, is a suitable chassis to synthesize endophenazine A. Herein, we designed an artificial biosynthetic pathway for the synthesis of endophenazine A in *P. chlororaphis* P3. Primarily, the prenyltransferase PpzP from *Streptomyces anulatus* 9663 was introduced into *P. chlororaphis* P3 and successfully synthesized endophenazine A. Another phenazine compound, endophenazine A1, was discovered and identified as a leakage of the intermediate 4-hydroxy-3-methyl-2-butene pyrophosphate (HMBPP). Finally, the yield of endophenazine A reached 279.43 mg/L, and the yield of endophenazine A1 reached 189.2 mg/L by metabolic engineering and medium optimization. In conclusion, we successfully synthesized endophenazine A and endophenazine A1 in *P. chlororaphis* P3 for the first time and achieved the highest titer, which provides a reference for the heterologous synthesis of terpenoid phenazines.

## 1. Introduction

Phenazines are a class of important nitrogen-containing heterocyclic compounds that are mainly secreted by *Pseudomonas* and *Streptomyces* [1]. Recently, they have attracted significant attention due to their widespread applications in agriculture, medicine, and industry [2,3,4,5]. Although phenazines can be chemically synthesized, these methods have many drawbacks, such as the generation of toxic by-products, low yields, and difficulty in purification [6,7]. Therefore, the biosynthesis of phenazines by green and environmentally friendly microbial fermentation is considered a promising choice.

**Figure 1:**
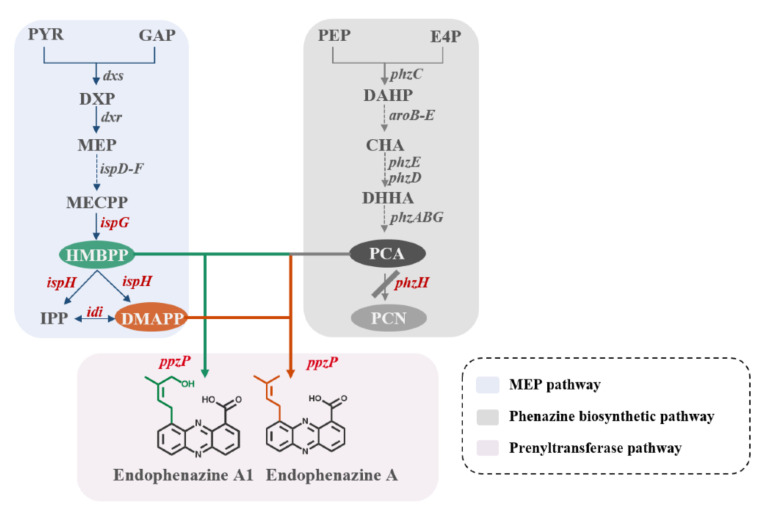
Design of the biosynthetic pathway for endophenazines in *Pseudomonas chlororaphis* P3. The *P. chlororaphis* endogenous MEP pathway is shown in blue, the *P. chlororaphis* phenazine synthesis pathway is shown in grey, and the endophenazines pathway from *S. anulatus* 9663 is shown in pink. Genes: *phzH*, encoding glutamine amidotransferase; *ispG*, encoding 4-hydroxy-3-methylbut-2-en-1-yl diphosphate synthase; *ispH*, encoding 4-hydroxy-3-methylbut-2-enyl diphosphate reductase; *idi*, encoding isopentenyl diphosphate isomerase; and *ppzP*, encoding prenyltransferase.

Terpenoid phenazines show antitumor and antibacterial activities and are potential cancer therapeutic agents (Table S1) [3,8,9,10,11]. Endophenazine A is a C-prenylation phenazine of PCA, catalyzed by prenyltransferase PpzP, and possesses good antibacterial activity [12,13] (Figure 1). PpzP expresses a soluble protein and belongs to the ABBA prenyltransferase family due to its special α-β-β-α structure. In the absence of magnesium or other divalent cations, PpzP is able to accept the isopentenyl donor from dimethylallyl pyrophosphate (DMAPP), connecting C-1 of DMAPP and C-9 of 5,10-dihydrophenazine 1-carboxylate (dihydro-PCA) to form endophenazine A [12]. Endophenazine A is mainly occurred in *Streptomyces*, but the yield is quite low [14]. Generally, *Streptomyces* synthesized phenazine compounds using phenazine-1,6-dicarboxylic acid (PDC) as a precursor, such as griseolutein acid [15,16] and lomofungin [17], which is not a precursor for the synthesis of endophenazine A. *Pseudomonas* mainly use phenazine-1-carboxylic acid (PCA) as a precursor to synthesize phenazine derivatives, such as phenazine-1-carboxamide (PCN), 2-hydroxyphenazine (2-OH-PHZ), 1-hydroxyphenazine (1-OH-PHZ), and pyocyanin (PYO) [18,19,20,21,22]. *Pseudomonas chlororaphis* P3 is a high-yielding mutagenic strain for PCN production with complete phenazine synthetic gene cluster *phzABCDEFG* and *phzH*, encoding glutamine amidotransferase [18,23,24,25], and can be transformed into a good host for the synthesis of terpenoid phenazines.

In addition to PCA, DMAPP is also an essential precursor for the biosynthesis of endophenazine A, which is produced via two pathways: the 2-methyl-D-erythritol-4-phosphate (MEP) pathway and the mevalonate (MVA) pathway. Consistent with most bacteria, *Pseudomonas* only possesses the MEP pathway [26]. The MEP pathway, called the non-mevalonate (MVA) pathway, begins with pyruvate and glyceraldehyde 3-phosphate (GAP), and followed by continuous enzyme catalysis to synthesize 2-C-methyl-D-erythritol 2, 4-cyclodiphosphate (MECPP) [27]. Next, MECPP is catalyzed by 4-hydroxy-3-methyl-2-butene pyrophosphate (HMBPP) synthase (IspG) to HMBPP, which is reduced to isopentenyl pyrophosphate (IPP) and DMAPP by HMBPP reductase (IspH) [28]. In the last step, IPP and DMAPP can be reversibly converted by isopentenyl diphosphate isomerase (IDI) [29] (Figure 1). Due to the limitation of isoprene precursors, many studies have improved the concentration of isoprene by increasing the expression of key enzymes. For example, overexpression of *idi* enhanced the production of lycopene and β-carotene up to 4.5 and 2.7 folds, respectively [30]. In addition, the role of genes *ispG* and *ispH* in the MEP pathway have been elucidated and could be the important rate-limiting steps to synthesize secondary metabolites [31].

Based on the metabolic network of the phenazine biosynthesis pathway and MEP pathway, we designed an artificial biosynthetic pathway of endophenazines and successfully synthesized endophenazine A and endophenazine A1 in *P. chlororaphis* P3 for the first time (Figure 1). It was found that endophenazine A1 was a leakage of the intermediate HMBPP. Moreover, the yield of endophenazine A and endophenazine A1 were improved through metabolic engineering and medium optimization.

## 2. Materials and Methods

### 2.1. Strains, Plasmids, and Culture Conditions

All strains and plasmids are shown in Table 1, and primers are listed in Table S2. *P. chlororaphis* P3 was obtained by chemical mutagenesis of *P. chlororaphis* HT66 with a high production of PCN [24]. *P. chlororaphis* strains were grown in 5 mL Luria-Bertani (LB) broth and shake flask fermented in 60 mL King’s B medium (KB) and Y medium (37.08 mL/L glycerol, 20 g/L tryptone, 25.03 g/L soy peptone) at 28 °C, 200 rpm.

### 2.2. DNA Manipulation

Genes were deleted or integrated into the genome with a non-scar homologous recombination strategy in *P. chlororaphis* P3. First, the upstream and downstream fragment sequence of the target gene were ligated to the pK18mobsacB plasmid that contains the sucrose-sensitive *sacB* gene. Second, the recombinant plasmids were transformed into S17-1 (λplr) followed by *P. chlororaphis* P3. Finally, the resulting mutant strains were verified by DNA sequencing. The codon-optimized *ppzP*, *ispG*, *ispH*, and *idi* genes were ligated to the pBBR1MCS plasmid containing the lac promoter and transferred into *P. chlororaphis* by electroporation. The function and accession numbers of the genes are listed in Table S3.

### 2.3. Analytical Procedures for Phenazine Derivatives

During the fermentation process, 1 mL culture broth was taken every 12 h until 72 h. The extraction method of phenazine derivatives was consistent with that described by Deng et al. [32]. The mobile phase was 1‰ formic acid water and methanol. The compounds were separated by the gradient elution method (0−4 min, 20% methanol; 4−30 min, 50–95% methanol; 30−35 min, 20% methanol) with detection 254 nm.

### 2.4. Purification and Structural Identification of Phenazine Derivatives

Precisely, 5 L fermentation supernatant containing the target compound was adjusted to pH 2.0–3.0 with 6 M HCl, extracted with ethyl acetate and concentrated by evaporation at 40 °C. The crude phenazine extract from the supernatant was dissolved in 5-mL methanol and then passed through the Sephadex LH-20 column. The column was eluted with 10% methanol to remove impurities and eluted with 30% methanol to obtain the target compound. The 30% methanol eluate was collected separately in different tubes and analyzed by HPLC, and the eluate containing the target compound was mixed together. The filtered sample was separated by analytical HPLC (Agilent 1260 LC) with Agilent Eclipse XDB-C18. The purified compound was freeze-dried to form a solid powder. The purified compound was characterized by nuclear magnetic resonance (NMR) spectroscopy in the Instrumental Analysis Centre of Shanghai Jiao Tong University.

### 2.5. Statistical Analysis

All statistical figures were drawn using Prism software (GraphPad Software, La Jolla, CA, USA). All data presented were the average of three biological replicates and shown as the mean ± standard deviation.

## 3. Results

### 3.1. Construction of Biosynthetic Pathway for Endophenazine A in P. chlororaphis P3

**Figure 2:**
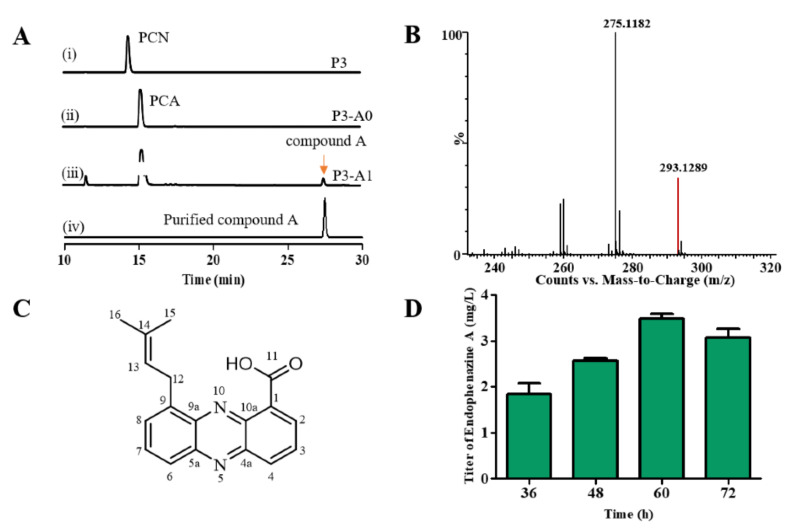
Endophenazine A was synthesized by *P. chlororaphis* with inserted *ppzP* gene. (**A**) HPLC profiles of P3, P3-A0, and P3-A1 strains fermentation broth and purified compound A. (**B**) The UPLC-MS result of compound A. (**C**) The structure of compound A. (**D**) The concentration of compound A in P3-A1 in different culture time. P3: a mutant from *P. chlororaphis* HT66; P3-A0: deletion of *phzH* in P3; P3-A1: introduction of *ppzP* in the chromosome of P3.

PCA is a precursor for the synthesis of endophenazine A, thus gene *phzH* in *P. chlororaphis* P3 was knocked out to get the strain P3-A0. Then, *ppzP* was selected to connect the phenazine biosynthetic and MEP pathways and inserted into the original *phzH* locus of the chromosome in P3-A0 to construct the P3-A1 strain. To detect whether new phenazines were synthesized, we extracted the phenazines from the fermentation broth of the strains P3, P3-A0, and P3-A1, and analyzed them with HPLC. As shown in Figure 2A, the deletion of the *phzH* gene led to the accumulation of PCA instead of PCN in P3-A0, and the insertion of the *ppzP* gene resulted in a new compound A in strain P3-A1. The extract of the fermentation broth was then purified by the preparative HPLC, and purified compound A was obtained. Subsequently, the purified compound A was analyzed by UPLC-MS and NMR. The m/z value of compound A was 293.1289 [M + H]^+^, suggesting that the molecular formula of compound A is C_18_H_16_N_2_O_2_ and the calculated value is 293.1285 (Figure 2B). The proton and carbon chemical shifts of compound A are shown in Figure S1 and Table S4. The ^1^H NMR (400 MHz, DMSO-*d6*) spectra showed peaks at δ 8.61 (d, *J =* 7.0 Hz, H2), 8.11 (d, *J =* 7.9 Hz, H3), 8.51 (d, *J =* 8.7 Hz, H4), 7.87 (d, *J =* 6.8 Hz, H6), 7.98 (d, *J =* 7.8 Hz, H7), 8.17 (d, *J =* 8.1 Hz, H8), 3.37 (br, H12), 5.51 (t, *J =* 7.3 Hz, H13), 1.79 (s, H15), and 1.73 (s, H16). The ^13^C NMR (100 MHz, DMSO-*d6*) spectra showed peaks at δ 138.8 (C1), 132.1 (C2), 127.6 (C3), 134.8 (C4), 142.5 (C4a), 143.7 (5a), 130.5 (C6), 133.9 (C7), 131.1 (C8), 139.3 (C9), 139.4 (C9a), 127.5 (C10a), 166.1 (C11), 29.3 (C12), 121.6 (C13), 133.6(C14), 25.5 (C15), and 17.8 (C16). According to these results, the structure of compound A was determined to be endophenazine A (Figure 2C).

### 3.2. Overexpression of ppzP Gene Enhanced the Production of Endophenazine A

**Figure 3:**
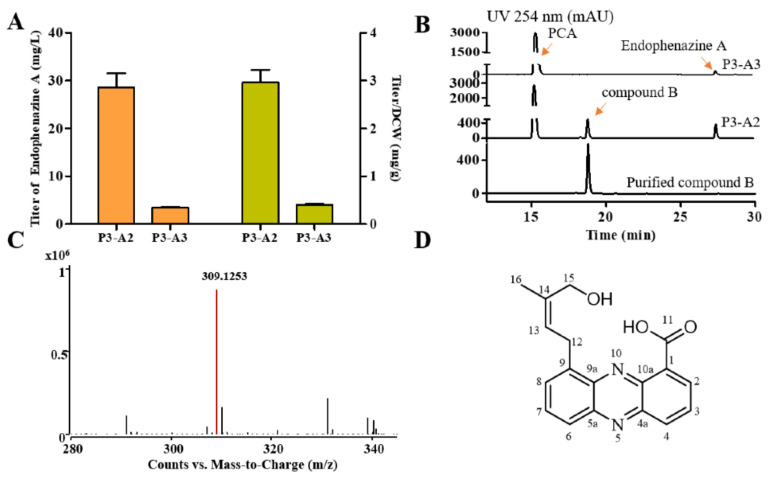
Influence of overexpression of *ppzP* on the titer of endophenazines. (**A**) The concentration of endophenazine A in strain P3-A2 and P3-A3. (**B**) HPLC profiles of P3-A2 and P3-A3 strains fermentation broth and purified compound B. (**C**) The UPLC-MS result of compound B. (**D**) The structure of compound B. P3-A2: overexpression of *ppzP* in P3-A1; P3-A3: integration of pBBR1MCS plasmid in P3-A1.

Although the strain P3-A1 synthesized endophenazine A, its highest titer was only 3.5 mg/L at 60 h (Figure 2D). Since there was only a single copy of the *ppzP* gene in the genome, prenyltransferase PpzP might be a rate-limiting step in synthesizing endophenazine A. Therefore, the *ppzP* gene was overexpressed under the control of lac promoter in P3-A1 to get the strain P3-A2, resulting in the titer of 28.64 mg/L endophenazine A, which was 7.17-times higher than that in the control strain P3-A3, and the titer/DCW of endophenazine A was increased from 0.40 mg/g in P3-A3 to 2.96 mg/g in P3-A2 (Figure 3A). This result indicated that *ppzP* is a rate-limiting step, and increasing its expression level can promote the production of endophenazine A.

### 3.3. Purification and Structural Identification of the Compound B

In addition to endophenazine A, another compound B in the strain P3-A2 was also detected (Figure 3B). Then, the purified compound B was analyzed by UPLC-MS and NMR. The m/z value of compound B is 309.1253 [M + H]^+^, suggesting that the molecular formula of compound B is C_18_H_16_N_2_O_3_ and the calculated value is 309.1234 (Figure 3C). The proton and carbon chemical shifts of compound B are shown in Figure S2 and Table S5. The ^1^H NMR (400 MHz, DMSO-*d6*) spectra showed peaks at δ 8.56 (dd, *J* = 7.0, 1.4 Hz, H2), 8.06 (dd, *J* = 8.7, 7.0 Hz, H3), 8.45 (dd, *J* = 8.7, 1.4 Hz, H4), 7.85 (dd, *J* = 6.8, 1.3 Hz, H6), 7.95 (dd, *J* = 8.8, 6.9 Hz, H7), 8.12 (dd, *J* = 8.7, 1.4 Hz, H8), 3.99 (d, *J* = 7.2 Hz H12), 5.71 (m, H13), 3.86 (s, H15), 4.68 (s, -OH15), and 1.76 (s, H16). The ^13^C NMR (100 MHz, DMSO-*d6*) spectra showed peaks at δ 137.9 (C1), 131.9 (C2), 127.4 (C3), 134.5 (C4), 142.2 (C4a), 144.3 (C5a), 130.4 (C6), 133.6 (C7), 130.9 (C8), 138.5 (C9), 139.1 (C9a), 127.4 (C10a), 166.0 (C11), 28.4 (C12), 120.2 (C13), 138.9(C14), 66.1 (C15), and 13.7 (C16). The structure of compound B was finally determined to be endophenazine A1 (Figure 3D).

### 3.4. HMBPP Is the Precursor for the Synthesis of Endophenazine A1

**Figure 4:**
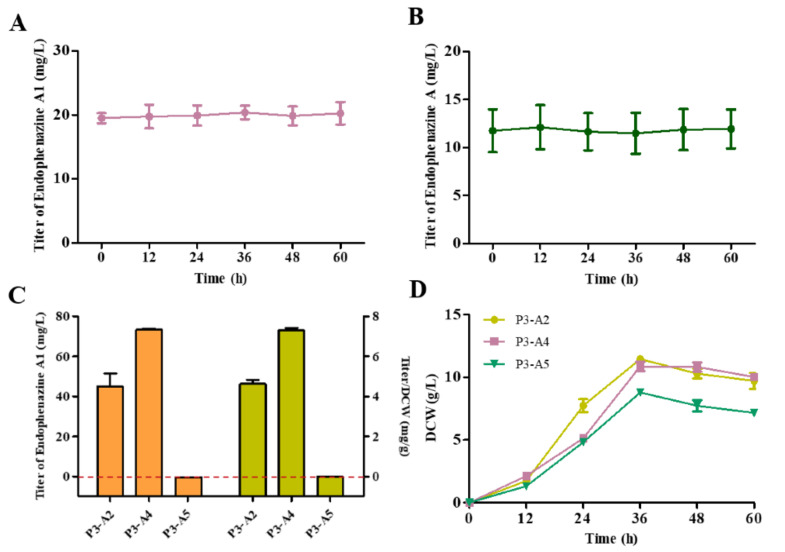
The synthesis of endophenazine A1 in *P. chlororaphis* P3. (**A**) The stability of endophenazine A1 in *P. chlororaphis* P3. (**B**) The stability of endophenazine A in *P. chlororaphis P3*. (**C**) The production of endophenazine A1 in strain P3-A2, P3-A4, and P3-A5. (**D**) The growth curve of P3-A2, P3-A4, and P3-A5. P3-A2: overexpression of *ppzP* in P3-A1*;* P3-A4: co-overexpression of *ppzP and ispG* in P3-A1; P3-A5: co-overexpression of *ppzP* and *ispH* in P3-A1.

Endophenazine A1 was first isolated from *Kitasatospora* sp. HKI 714 [33], but its synthesis mechanism has not been elucidated. Since endophenazine A and endophenazine A1 are similar in structure, we first assumed that endophenazine A1 is an intermediate, which can be further reduced to endophenazine A, or endophenazine A might be unstable and can be oxidized to endophenazine A1. To test this hypothesis, we cultured strain P3-A0 in KB medium containing endophenazine A and endophenazine A1, respectively. However, the concentration of endophenazine A and endophenazine A1 did not decrease significantly after 60 h of incubation (Figure 4A,B). These results showed that endophenazine A1 and endophenazine A cannot interconvert in *P. chlororaphis* P3. Our other assumption was that the leakage of intermediate might cause endophenazine A1. In the MEP pathway, the intermediate HMBPP is almost the same as the isoprene unit of the endophenazine A1, so HMBPP may be the precursor of endophenazine A1. In order to investigate the effect of HMBPP concentration on the production of endophenazine A1, we co-expressed *ispG* and *ispH* with *ppzP* to obtain strains P3-A4 and P3-A5, respectively. Compared with strain P3-A2, the concentration of endophenazine A1 in strain P3-A4 increased by 62%, and the titer/DCW of endophenazine A1 was increased from 4.63 mg/g in P3-A2 to 7.32 mg/g in P3-A4. On the contrary, strain P3-A5 hardly synthesized endophenazine A1 (Figure 4C). The overexpression of the *ispG* gene had less effect on cell growth, while overexpression of the *ispH* gene had a significant inhibition on cell growth (Figure 4D). The above results revealed that HMBPP is the precursor for the synthesis of endophenazine A1, which provides different metabolic engineering strategies for subsequent improvement in the production of endophenazine A and endophenazine A1.

### 3.5. Modification of Metabolic Pathways to Increase the Production of Endophenazine A

**Figure 5:**
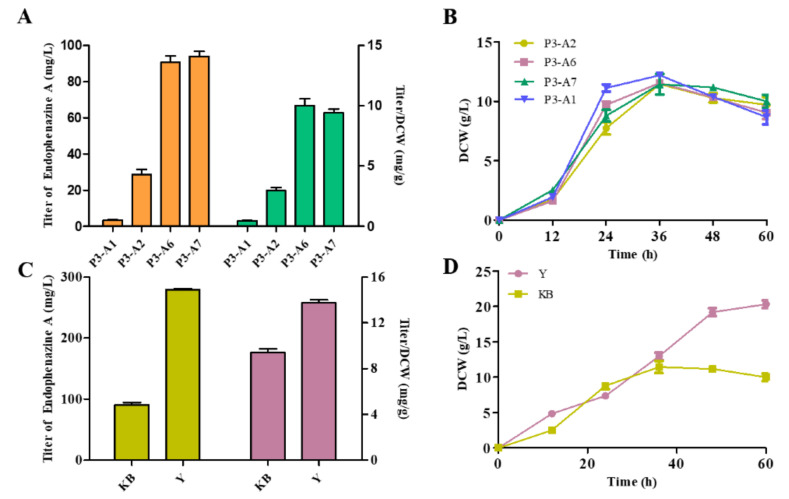
Effects of metabolic regulation and medium optimization on endophenazine A concentration (**A**,**C**) and cell growth (**B**,**D**) in *P. chlororaphis P3*. P3-A1: introduction of *ppzP* in the chromosome of P3; P3-A2: overexpression of *ppzP* in P3-A1*;* P3-A6: co-overexpression of *ppzP* and *idi* in P3-A1; P3-A7: co-overexpression of *ppzP*, *idi*, and *ispH* in P3-A1.

To improve the production of endophenazine A, a rational strategy was designed to drive the carbon flux to the biosynthesis of endophenazine A. Firstly, the rate-limiting gene *idi* and *ppzP* was co-expressed in P3-A1 to get the strain P3-A6. As seen in Figure 5A, the production of endophenazine A in P3-A6 was 90.61 mg/L, which was 24.89 times higher than that in P3-A1, and the titer/DCW of endophenazine A was increased from 0.47 mg/g in P3-A1 to 10.04 mg/g in P3-A6. To minimize the leakage of HMBPP, *ispH* was co-expressed with *ppzP* and *idi* in strain P3-A1 obtaining the strain P3-A7. The production of endophenazine A was 94.14 mg/L in P3-A7, which is 3.9% higher than P3-A6 (Figure 5A). The overexpression of these genes had no significant effect on cell growth (Figure 5B). To further increase the production of endophenazine A, P3-A7 was cultured in the optimized Y medium, in which the concentration of endophenazine A was 279.43 mg/L, and the titer/DCW of endophenazine A was increased from 9.42 mg/g in KB medium to 13.75 mg/g in KB medium (Figure 5C). The reason may be that rich nutrients lead to high biomass (Figure 5D).

### 3.6. Modification of Metabolic Pathways to Increase the Production of Endophenazine A1

**Figure 6:**
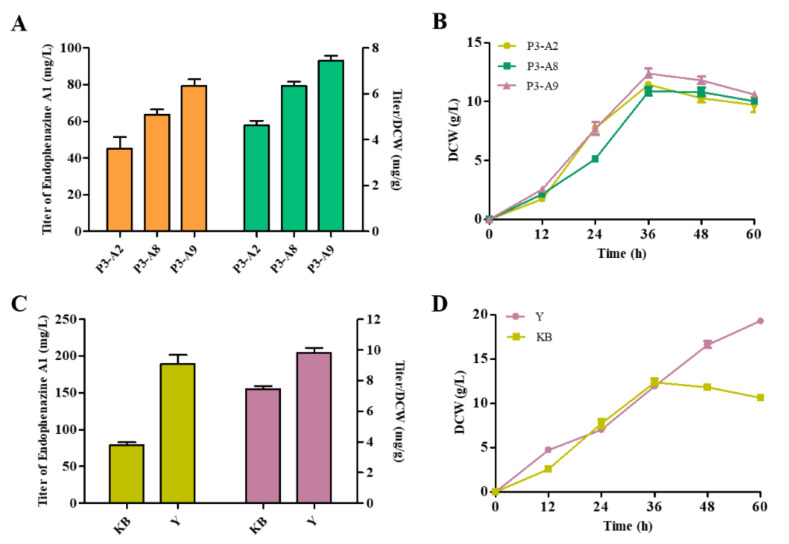
Effects of metabolic regulation and medium optimization on endophenazine A1 production (**A**,**C**) and cell growth (**B**,**D**) in *P. chlororaphis P3*. P3-A2: overexpression of *ppzP* in P3-A1*;* P3-A8: deletion of *idi* in P3-A2; P3-A9: co-overexpression of *ppzP* and *ispG* in P3-A8.

It has been proven that endophenazine A1 was a shunt pathway of endophenazine A. Therefore, we attempted to increase the production of endophenazine A1 by reducing the consumption of HMBPP and promoting the synthesis of HMBPP. The *ispH* gene is known to be necessary for bacterial growth and survival and cannot be deleted [34]. Therefore, we could not delete the *ispH* gene to block carbon flux from HMBPP to DMAPP. Previous results proved that overexpression of the *idi* gene promoted the flow of carbon flux to produce DMAPP, so we knocked out the *idi* gene in strain P3-A2 and obtained the strain P3-A8 to reduce the accumulation of DMAPP and increase the accumulation of HMBPP. As shown in Figure 6A, the production of endophenazine A1 in P3-A8 was 63.6 mg/L, which increased by 41% compared with P3-A2, and the titer/DCW of endophenazine A1 was increased from 4.63 mg/g in P3-A2 to 6.34 mg/g in P3-A8. Then, in order to increase the supply of precursor HMBPP, the *ispG* gene was co-overexpressed with *ppzp* in strain P3-A8 to get P3-A9, resulting in the production titer of 79.24 mg/L endophenazine A1, which was 24% higher than that of strain P3-A8, and the titer/DCW of endophenazine A1 was increased from 6.34 mg/g in P3-A8 to 7.45 mg/g in P3-A9 (Figure 6A). At the same time, these gene manipulations had no significant effect on cell growth (Figure 6B). Finally, the titer of endophenazine A1 in the Y medium of strain P3-A9 increased to 189.2 mg/L, which was 2.4 folds in the KB medium, and the titer/DCW of endophenazine A1 was increased from 7.45 mg/g in KB medium to 9.81 mg/g in KB medium (Figure 6C). The reason may be similar to the high production of endophenazine A (Figure 6D).

## 4. Discussion

Endophenazine A is a C-prenylation phenazine catalyzed by prenyltransferases PpzP from *S. anulatus* 9663 or EpzP from *S. cinnamonensis* DSM 1042; the conversion rate of PpzP is higher than that of EpzP10 [35]. Although the production of endophenazine A was not reported in these two *Streptomyces*, the highest yield of endophenazine A was achieved in the engineered *S. coelicolor* M512, which heterologously expressed the whole gene cluster of endophenazine was only 20 mg/L [14]. Due to the fast growth and clear genetic background of *P. chlororaphis* P3 [36], the high yield of precursor compound PCA and endogenous MEP pathway, we designed an artificial biosynthetic pathway to produce endophenazine A in *P. chlororaphis* P3.

Besides endophenazine A, endophenazine A1 was synthesized (Figure 3B). Daniel et al. isolated endophenazine A1 in *Kitasatospora* sp. HKI 714, which showed antibacterial activity against *Mycobacterium fortuitum* and *Mycobacterium aureus* [33]. Later, it was reported that endophenazine A1 and endophenazine A were also detected in *Kitasatospora* sp. MBT66 [37,38]. However, the synthesis mechanism of endophenazine A1 was unclear. In this study, we found that endophenazine A1 and endophenazine A are relatively stable in the culture of *P. chlororaphis* P3 and cannot be converted to each other, indicating that endophenazine A1 is not an intermediate in the synthesis of endophenazine A and endophenazine A cannot be oxidized to form endophenazine A1 (Figure 4A,B). Then, we found that overexpression of the *ispG* gene enhanced the accumulation of endophenazine A1, while overexpression of the *ispH* gene reduced the production of endophenazine A1 (Figure 4C). These results are consistent with previous studies in which the overexpression of the *ispG* gene led to the enhancement of HMBPP, while overexpression of the *ispH* gene led to the reduction of HMBPP [31]. These results revealed that HMBPP is a key switch to control the metabolic flux toward endophenazine A and shunt products endophenazine A1.

**Figure 7:**
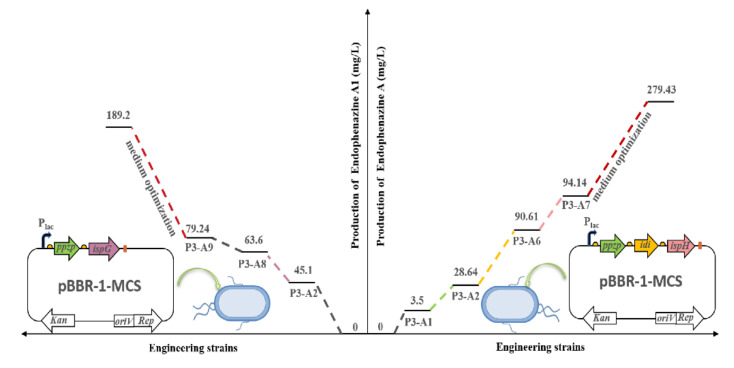
Summary of metabolic engineering of *P. chlororaphis P3* and medium optimization for stepwise increasing the titer of endophenazines. Left, concentration of endophenazine A1. Right, concentration of endophenazine A. P3-A1: introduction of *ppzP* in the chromosome of P3; P3-A2: overexpression of *ppzP* in P3-A1*;* P3-A3: integration of pBBR1MCS plasmid in P3-A1; P3-A4: co-overexpression of *ppzP and ispG* in P3-A1; P3-A5: co-overexpression of *ppzP* and *ispH* in P3-A1; P3-A6: co-overexpression of *ppzP* and *idi* in P3-A1; P3-A7: co-overexpression of *ppzP*, *idi*, and *ispH* in P3-A1; P3-A8: deletion of *idi* in P3-A2; P3-A9: co-overexpression of *ppzP* and *ispG* in P3-A8.

Accordingly, we used metabolic engineering strategies to regulate the carbon flow of DMAPP and HMBP to achieve high production of endophenazine A and endophenazine A1, respectively. Firstly, we overexpressed the key genes of *ppzP*, *idi*, and *ispH* of the endophenazine A pathway in P3-A1, and the production of endophenazine A was significantly increased (Figure 5A). This result is consistent with the previous report, where the *ispH* gene catalyzed HMBPP into IPP and DMAPP at a ratio of 6.3:1, but it reduced up to 1:3.1 after treatment with gene *idi* [39]. Subsequently, the *ispG* and *ppzP* genes were co-overexpressed in P3-A8, and the concentration of endophenazine A1 increased by 80% (Figure 6A). This result is consistent with previous studies in which the overexpression of the *ispG* gene led to the enhancement of HMBPP. Finally, the concentration of endophenazine A and endophenazine A1 in the engineered *P. chlororaphis* P3 was 279.43 mg/L and 189.2 mg/L, respectively, which were remarkably enhanced with the stepwise metabolic engineering and medium optimization (Figure 7).

Protein engineering is a widely used effective strategy to improve enzyme-substrate specificity and catalytic efficiency. In the endophenazine A biosynthesis pathway, the catalytic efficiency of EpzP is 14 times higher than that of the wild-type enzyme through rational design [40]. Edward et al. realized the conversion of transketolase substrate specificity to fatty aldehyde through directed evolution [41]. This is a reference to increase the yield and diversity of terpenoid phenazines in the future.

## 5. Conclusions

In this study, two terpenoid phenazine pathways were designed and constructed in *P. chlororaphis* P3, resulting in the production of 279.43 mg/L endophenazine A and 189.2 mg/L endophenazine A1, respectively, through rational metabolic engineering strategies, which is the highest titers in microbial production of endophenazine A and endophenazine A1 up to now. This study enriched the diversity of phenazine compounds synthesized by *P. chlororaphis* P3 and laid the foundation for the future synthesis and large-scale production of other terpenoid phenazines.

## Figures and Tables

**Table 1:** Strains and plasmids used in this study.

**Strains/Plasmids**	**Characteristics**	**Reference**
Strains		
DH5α	The host for plasmid transformation	Lab stock
S17-1(λpir)	Donor strain for conjugation	Lab stock
P3	A mutant from *P. chlororaphis* HT66 with a high PCN	Lab stock
P3-A0 P3-A1	Deletion of *phzH* in P3 Introduction of *ppzP* in the chromosome of P3	This study This study
P3-A2 P3-A3 P3-A4	Overexpression of *ppzP* in P3-A1 Integration of pBBR1MCS plasmid in P3-A1 Co-overexpression of *ppzP and ispG* in P3-A1	This study This study This study
P3-A5	Co-overexpression of *ppzP* and *ispH* in P3-A1	This study
P3-A6	Co-overexpression of *ppzP* and *idi* in P3-A1	This study
P3-A7 P3-A8 P3-A9	Co-overexpression of *ppzP,idi* and *ispH* in P3-A1 Deletion of *idi* in P3-A2 Overexpression of *ispG* in P3-A8	This study This study This study
Plasmids		
pK18*mobsacB*	Broad-host-range plasmid for gene deletion, *sacB*, Kan^r^	Lab stock
pK18-*phzH* pK18-*idi*	pK18*mobsacB* plasmid for *phzH* deletion pK18*mobsacB* plasmid for *idi* deletion	This study This study
pBBR1MCS	Overexpression vector with *lac* promoter, Kan^r^	Lab stock
pBBR-*ppzP*	pBBR1MCS for overexpression *ppzP*	This study
pBBR-*ppzP*-*idi*	pBBR1MCS for co-overexpression *ppzP* and *idi*	This study
pBBR-*ppzP*-*ispG* pBBR- *ppzP-ispH* pBBR-*ppzP-idi-ispH*	pBBR1MCS for co-overexpression *ppzP* and *ispG* pBBR1MCS for co-overexpression *ppzP* and *ispH* pBBR1MCS for co-overexpression *ppzP*, *idi* and *ispH*	This study This study This study

Kan^r^ represent kanamycin.

## Data Availability

Not applicable.

## Supplementary Materials

The following supporting information can be downloaded at: https://www.mdpi.com/article/10.3390/biology11030363/s1, Figure S1: ^1^H NMR (a) and ^13^C NMR (b) spectra of compound A, Figure S2: ^1^H NMR (a) and ^13^C NMR (b) spectra of compound B; Table S1: Terpenoid phenazines, Table S2: The primers sequence used in this study, Table S3: The function and accession numbers of the genes, Table S4: ^1^H and ^13^C NMR spectral data of compound A in DMSO-*d6*, Table S5: ^1^H and ^13^C NMR spectral data of compound B in DMSO-*d6*. References in the supporting information are listed in the manuscript.

## References

[1] Mavrodi D.V., Peever T.L., Mavrodi O.V., Parejko J.A., Raaijmakers J.M., Lemanceau P., Mazurier S., Heide L., Blankenfeldt W., Weller D.M. (2010). Diversity and evolution of the phenazine biosynthesis pathway. Appl. Environ. Microbiol..

[2] Pierson L.S., Pierson E.A. (2010). Metabolism and function of phenazines in bacteria: Impacts on the behavior of bacteria in the environment and biotechnological processes. Appl. Microbiol. Biotechnol..

[3] Laursen J.B., Nielsen J. (2004). Phenazine natural products: Biosynthesis, synthetic analogues, and biological activity. Chem. Rev..

[4] Ligon J.M., Hill D.S., Hammer P.E., Torkewitz N.R., Hofmann D., Kempf H.J., Pée K.H.v. (2000). Natural products with antifungal activity from *Pseudomonas biocontrol* bacteria. Pest Manag. Sci..

[5] Makgatho M.E., Anderson R., O’Sullivan J.F., Egan T.J., Freese J.A., Cornelius N., van Rensburg C.E. (2000). Tetramethylpiperidine-substituted phenazines as novel anti-plasmodial agents. Drug Dev. Res..

[6] Cheluvappa R. (2014). Standardized chemical synthesis of Pseudomonas aeruginosa pyocyanin. MethodsX.

[7] Elhady H.A., El-Mekawy R.E., Fadda A. (2020). Valuable Chemistry of Phenazine Derivatives: Synthesis, Reactions and, Applications. Polycycl. Aromat. Compd..

[8] Kondratyuk T.P., Park E.-J., Yu R., Van Breemen R.B., Asolkar R.N., Murphy B.T., Fenical W., Pezzuto J.M. (2012). Novel marine phenazines as potential cancer chemopreventive and anti-inflammatory agents. Mar. Drugs.

[9] Cimmino A., Evidente A., Mathieu V., Andolfi A., Lefranc F., Kornienko A., Kiss R. (2012). Phenazines and cancer. Nat. Prod. Rep..

[10] Gebhardt K., Schimana J., Krastel P., Dettner K., Rheinheimer J., Zeeck A., Fiedler H.-P. (2002). Endophenazines A–D, New Phenazine Antibiotics from the Arthropod Associated Endosymbiont *Streptomyces anulatus* I. Taxonomy, Fermentation, Isolation and Biological Activities. J. Antibiot..

[11] Omura S., Eda S., Funayama S., Komiyama K., Takahashi Y., Woodruff H.B. (1989). Studies on a novel antitumor antibiotic, phenazinomycin: Taxonomy, fermentation, isolation, and physicochemical and biological characteristics. J. Antibiot..

[12] Saleh O., Gust B., Boll B., Fiedler H.-P., Heide L. (2009). Aromatic prenylation in phenazine biosynthesis: Dihydrophenazine-1-carboxylate dimethylallyltransferase from *Streptomyces anulatus*. J. Biol. Chem..

[13] Krastel P., Zeeck A., Gebhardt K., Fiedler H.-P., Rheinheimer J. (2002). Endophenazines A–D, new phenazine antibiotics from the athropod associated endosymbiont *Streptomyces anulatus* II. Structure elucidation. J. Antibiot..

[14] Saleh O., Flinspach K., Westrich L., Kulik A., Gust B., Fiedler H.-P., Heide L. (2012). Mutational analysis of a phenazine biosynthetic gene cluster in *Streptomyces anulatus* 9663. Beilstein J. Org. Chem..

[15] Giddens S.R., Feng Y., Mahanty H.K. (2002). Characterization of a novel phenazine antibiotic gene cluster in *Erwinia herbicola* Eh1087. Mol. Microbiol..

[16] Shi Y.-M., Brachmann A.O., Westphalen M.A., Neubacher N., Tobias N.J., Bode H.B. (2019). Dual phenazine gene clusters enable diversification during biosynthesis. Nat. Chem. Biol..

[17] Zhang C., Sheng C., Wang W., Hu H., Peng H., Zhang X. (2015). Identification of the lomofungin biosynthesis gene cluster and associated flavin-dependent monooxygenase gene in *Streptomyces lomondensis* S015. PLoS ONE.

[18] Chin-A-Woeng T.F., Thomas-Oates J.E., Lugtenberg B.J., Bloemberg G.V. (2001). Introduction of the *phzH* gene of *Pseudomonas chlororaphis* PCL1391 extends the range of biocontrol ability of phenazine-1-carboxylic acid-producing *Pseudomonas* spp. strains. Mol. Plant-Microbe Interact..

[19] Delaney S.M., Mavrodi D.V., Bonsall R.F., Thomashow L.S. (2001). *phzO*, a gene for biosynthesis of 2-hydroxylated phenazine compounds in *Pseudomonas aureofaciens* 30-84. J. Bacteriol..

[20] Greenhagen B.T., Shi K., Robinson H., Gamage S., Bera A.K., Ladner J.E., Parsons J.F. (2008). Crystal structure of the pyocyanin biosynthetic protein PhzS. Biochemistry.

[21] Parsons J.F., Greenhagen B.T., Shi K., Calabrese K., Robinson H., Ladner J.E. (2007). Structural and functional analysis of the pyocyanin biosynthetic protein PhzM from *Pseudomonas aeruginosa*. Biochemistry.

[22] Mavrodi D.V., Blankenfeldt W., Thomashow L.S. (2006). Phenazine compounds in fluorescent *Pseudomonas* spp. biosynthesis and regulation. Annu. Rev. Phytopathol..

[23] Peng H., Zhang P., Bilal M., Wang W., Hu H., Zhang X. (2018). Enhanced biosynthesis of phenazine-1-carboxamide by engineered *Pseudomonas chlororaphis* HT66. Microb. Cell Fact..

[24] Jin X.-J., Peng H.-S., Hu H.-B., Huang X.-Q., Wang W., Zhang X.-H. (2016). iTRAQ-based quantitative proteomic analysis reveals potential factors associated with the enhancement of phenazine-1-carboxamide production in *Pseudomonas chlororaphis* P3. Sci. Rep..

[25] Mavrodi D.V., Ksenzenko V.N., Bonsall R.F., Cook R.J., Boronin A.M., Thomashow L.S. (1998). A seven-gene locus for synthesis of phenazine1-carboxylic acid by *Pseudomonas fluorescens 2-79*. J. Bacteriol..

[26] Hernandez-Arranz S., Perez-Gil J., Marshall-Sabey D., Rodriguez-Concepcion M. (2019). Engineering *Pseudomonas putida* for isoprenoid production by manipulating endogenous and shunt pathways supplying precursors. Microb. Cell Fact..

[27] Sharkey T.D., Wiberley A.E., Donohue A.R. (2008). Isoprene emission from plants: Why and how. Ann. Bot..

[28] Zhao L., Chang W.-C., Xiao Y., Liu H.-W., Liu P. (2013). Methylerythritol phosphate pathway of isoprenoid biosynthesis. Annu. Rev. Biochem..

[29] Banerjee A., Sharkey T. (2014). Methylerythritol 4-phosphate (MEP) pathway metabolic regulation. Nat. Prod. Rep..

[30] Albrecht M., Misawa N., Sandmann G. (1999). Metabolic engineering of the terpenoid biosynthetic pathway of *Escherichia coli* for production of the carotenoids β-carotene and zeaxanthin. Biotechnol. Lett..

[31] Li Q., Fan F., Gao X., Yang C., Bi C., Tang J., Liu T., Zhang X. (2017). Balanced activation of IspG and IspH to eliminate MEP intermediate accumulation and improve isoprenoids production in *Escherichia coli*. Metab. Eng..

[32] Deng R.X., Zhang Z., Li H.L., Wang W., Hu H.B., Zhang X.H. (2021). Identification of a Novel Bioactive Phenazine Derivative and Regulation of *phoP* on Its Production in *Streptomyces lomondensis* S015. J. Agric. Food Chem..

[33] Heine D., Martin K., Hertweck C. (2014). Genomics-guided discovery of endophenazines from *Kitasatospora* sp. HKI 714. J. Nat. Prod..

[34] McAteer S., Coulson A., McLennan N., Masters M. (2001). The *lytB* gene of *Escherichia coli* is essential and specifies a product needed for isoprenoid biosynthesis. J. Bacteriol..

[35] Seeger K., Flinspach K., Haug-Schifferdecker E., Kulik A., Gust B., Fiedler H.P., Heide L. (2011). The biosynthetic genes for prenylated phenazines are located at two different chromosomal loci of *Streptomyces cinnamonensis* DSM 1042. Microb. Biotechnol..

[36] Wang S., Fu C., Bilal M., Hu H., Wang W., Zhang X. (2018). Enhanced biosynthesis of arbutin by engineering shikimate pathway in *Pseudomonas chlororaphis* P3. Microb. Cell Fact..

[37] Wu C., Van Wezel G.P., Choi Y.H. (2015). Identification of novel endophenaside antibiotics produced by *Kitasatospora* sp. MBT66. J. Antibiot..

[38] Wu C., Medema M.H., Läkamp R.M., Zhang L., Dorrestein P.C., Choi Y.H., Van Wezel G.P. (2016). Leucanicidin and endophenasides result from methyl-rhamnosylation by the same tailoring enzymes in *Kitasatospora* sp. MBT66. ACS Chem. Biol..

[39] Gräwert T., Kaiser J., Zepeck F., Laupitz R., Hecht S., Amslinger S., Schramek N., Schleicher E., Weber S., Haslbeck M. (2004). IspH protein of *Escherichia coli*: Studies on Iron− Sulfur cluster implementation and catalysis. J. Am. Chem. Soc..

[40] Zocher G., Saleh O., Heim J.B., Herbst D.A., Heide L., Stehle T. (2012). Structure-based engineering increased the catalytic turnover rate of a novel phenazine prenyltransferase. PLoS ONE.

[41] Hibbert E.G., Senussi T., Smith M.E., Costelloe S.J., Ward J.M., Hailes H.C., Dalby P.A. (2008). Directed evolution of transketolase substrate specificity towards an aliphatic aldehyde. J. Biotechnol..

