# Supplementary material for: Development of Artificial Synthetic Pathway of Endophenazines in Pseudomonas chlororaphis P3
Source: Biology (Basel). 2022 Feb 24;11(3):363. doi: 10.3390/biology11030363 (PMC8945225; doi:10.3390/biology11030363)
Supplement: Supplementary file 1 [file biology-11-00363-s001.zip › biology-1586120-supplementary.pdf]

Submitted to Biology

Supporting Information for

Development of Artificial Synthetic Pathway of Endophena-zines in *Pseudomonas chlororaphis* P3

Ying Liu[1], Shengjie Yue[1], Muhammad Bilal[2], Malik Jan[1], Wei Wang[1,3], Hongbo Hu[1,3], and Xuehong Zhang[1,3]*

[1] State Key Laboratory of Microbial Metabolism, School of Life Sciences and Biotechnology, Shanghai Jiao Tong University, Shanghai 200240, China; liuyinger@sjtu.edu.cn (Y.L.); yuesj_sjtu@163.com (S.Y.); jan.malik@sjtu.edu.cn (M.J.); weiwang100@sjtu.edu.cn (W.W.); hbhu@sjtu.edu.cn (H.H.)

[2] School of Life Science and Food Engineering, Huaiyin Institute of Technology , Huaian 223003, China; bilaluaf@hyit.edu.cn (M.B.)

[3] Shanghai Jiao Tong University-Shanghai Nongle Joint R&D Center on Biopesticides and Biofertilizers，Shanghai 200240, China

*Correspondence: xuehzhang@sjtu.edu.cn; Tel.: +86-21-3420-6742

Figure captions

Figure S1. ^{1}H NMR (a) and ^{13}C NMR (b) spectra of compound A.

Figure S2. ^{1}H NMR (a) and ^{13}C NMR (b) spectra of compound B.

Table captions

Table S1. Terpenoid phenazines.

Table S2. The primers sequence used in this study.

Table S3. The function and accession numbers of genes.

Table S4. ^{1}H and ^{13}C NMR spectral data of compound A in DMSO-*d6*.

Table S5. ^{1}H and ^{13}C NMR spectral data of compound B in DMSO-*d6*.

Figure S1. ^{1}H NMR (a) and ^{13}C NMR (b) spectra of compound A.

a

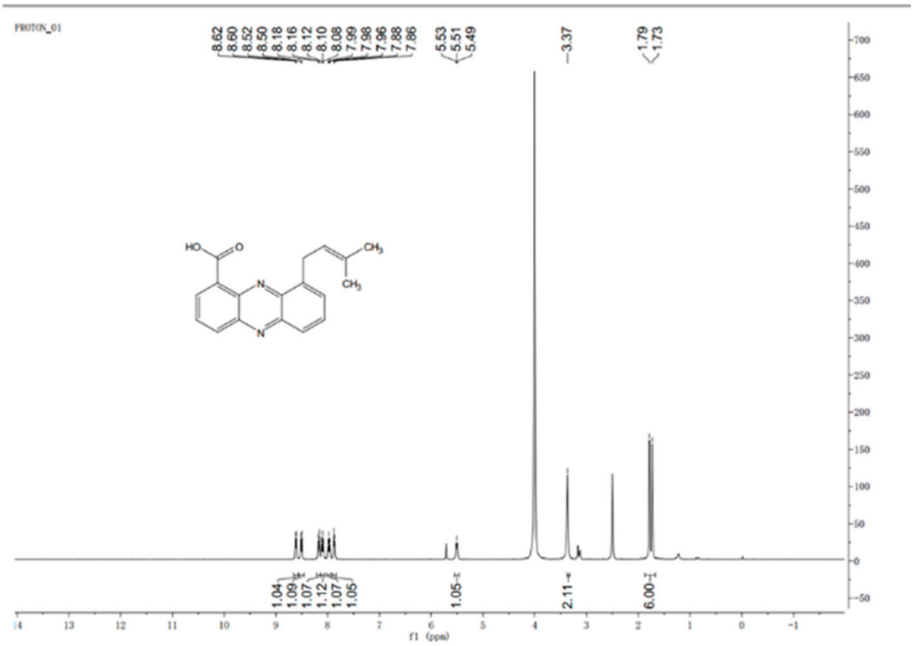

b

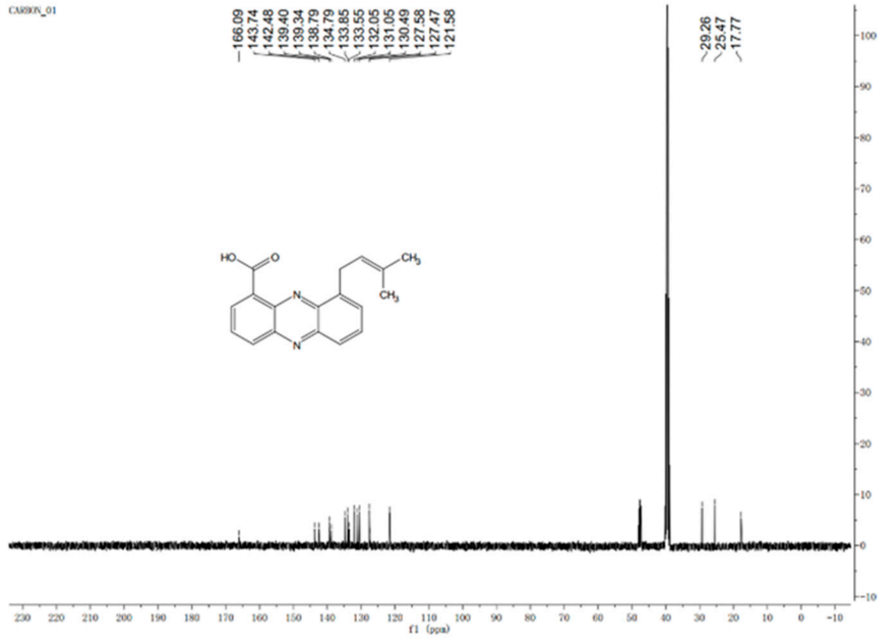

Figure S2. ^{1}H NMR (a) and ^{13}C NMR (b) spectra of compound B.

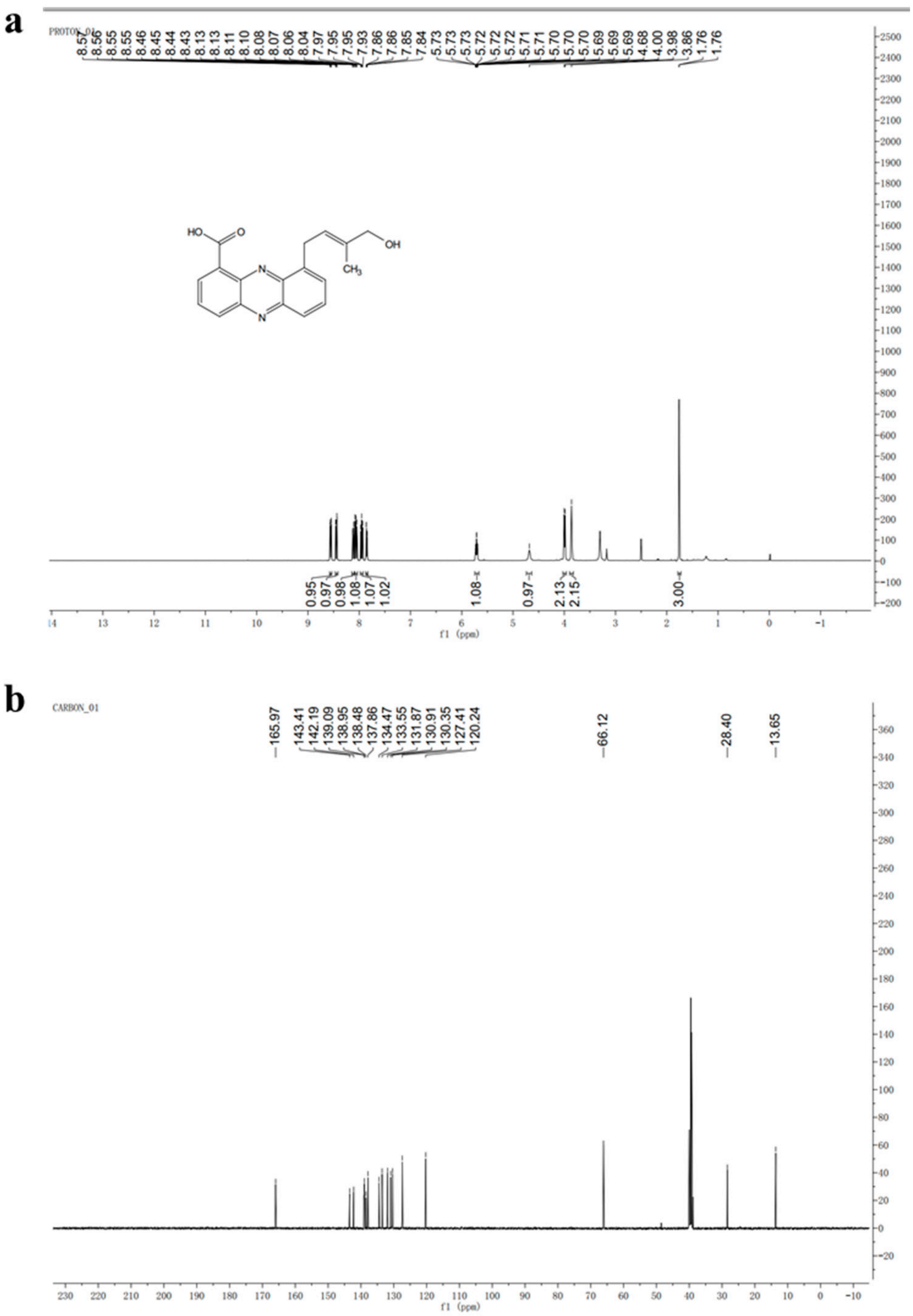

Table S1. Terpenoid phenazines.

Structure/Name	Activities	Compound source
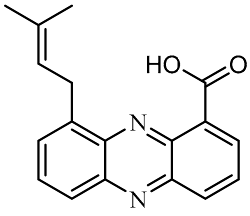 Endophenazine A	Exhibited activity against Gram-positive bacteria and some fungi	*S. anulatus* [10]
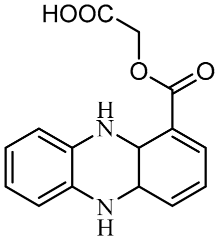 Endophenazine D	Exhibited activity against Gram-positive bacteria and some fungi	*S. anulatus* [10]
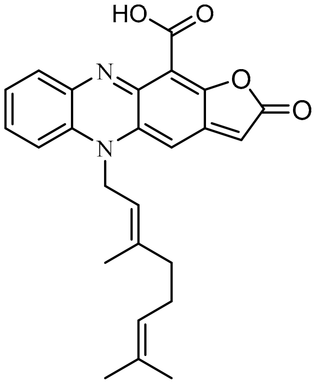 Benthocyanin B	Inhibits hemolysis of rat erythrocytes	*S. prunicolor* [3]
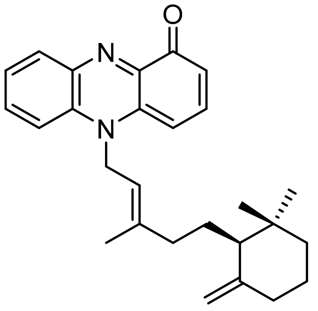 Phenazinomycin	Showed activity against murine tumors	*Streptomyces* sp. WK-2057 [11]

Table S2. The primers sequence used in this study.

Primers	Primer sequences (5'→3')	
phzH-F1	GACATGATTACGAATTCAAGCCGCTGTTGGGTAAAGG	
phzH-R1	TTAGTAACTCCTGTAATTATTCATT	
phzH-F2	ATTACAGGAGTTACTAATACGAGCCTGAGGGAGCCAC	*phzH* deletion
phzH-R2	CGGCCAGTGCCAAGCTTGCCATCGGCATTCAGGAAGTAG	
idi-F1	TATGACATGATTACGAATTCTCATCAGAAAGAACTATCCCACC	
idi-R1	GATGCTTCCGAAACTCCCT	
idi-F2	CAGGGAGTTTCGGAAGCATCGAGCATCCGTACGACAGACC	*idi* deletion
idi-R2	CGACGGCCAGTGCCAAGCTTGACATCGCTCACGAACTGC	
ppzP-sF	GGGAACAAAAGCTGGGTACCGATGAGCGAATCGGCCGAGCT	
ppzP-sR	TCGATACCGTCGACCTCGAGTCAGCCGGCGTCCGCGGTCA	single gene overexpression
idi-sF	GGGAACAAAAGCTGGGTACCGATGGAAGAAACCCTTATCCTGGT	
idi-sR	TCGATACCGTCGACCTCGAGTCATCCATGACTAGAAGAGCTT	
ppzP-dF	GGGAACAAAAGCTGGGTACCGATGAGCGAATCGGCCGAGCT	
ppzP-dR	TCAGCCGGCGTCCGCGGTCA	
idi-dF	ACCGCGGACGCCGGCTGAAGGAGGATGCCGCCATGGAAGAAACCCTTATCCTGGT	
idi-dR	CTCTAGAACTAGTGGATCCTCATCCATGACTAGAAGAGCTT	
ispG-dF	ACCGCGGACGCCGGCTGAAGGAGGATGCCGCCATGCACGGCGAATCTCCAA	double gene co-overexpression
ispG-dR	CTCTAGAACTAGTGGATCCTTAGCCGCGCGCGATCAGCG	
ispH-dF	ACCGCGGACGCCGGCTGAAGGAGGATGCCGCCATGCAAATCAAACTCGCCAACC	
ispH-dR	CGGTGGCGGCCGCTCTAGATCAGAGCAGCGAACGAACGC	

Table S3. The function and accession numbers of genes.

Protein	Function	Accession numbers
PhzH	Glutamine amidotransferase	WP_025806287.1
PpzP	Prenyltransferase	CAX48655.1
IspG	4-hydroxy-3-methylbut-2-en-1-yl diphosphate synthase	WP_007929536.1
IspH	4-hydroxy-3-methylbut-2-enyl diphosphate reductase	WP_025807544.1
Idi	Isopentenyl diphosphate isomerase	WP_025810178.1

Table S4. ^1H and ^{13}C NMR spectral data of compound A in DMSO-*d6*

position	δ_H (J in Hz)	δ_C (ppm)
1	--	138.8
2	8.61, d, (7.0)	132.1
3	8.11, d, (7.9)	127.6
4	8.51, d, (8.7)	134.8
4a	--	142.5
5	--	--
5a	--	143.7
6	7.87, d, (6.8)	130.5
7	7.98, d, (7.8)	133.9
8	8.17, d, (8.1)	131.1
9	--	139.3
9a	--	139.4
10	--	--
10a	--	127.5
11	--	166.1
12	3.37, br	29.3
13	5.51, t, (7.3)	121.6
14	--	133.6
15	1.79, s	25.5
16	1.73, s	17.8

Table S5. ^{1}H and ^{13}C NMR spectral data of compound B in DMSO-*d6*

position	δ_H (J in Hz)	δ_C (ppm)
1	--	137.9
2	8.56, dd, (7.0/1.4)	131.9
3	8.06, dd, (8.7/7.0)	127.4
4	8.45, dd, (8.7/1.4)	134.5
4a	--	142.2
5	--	--
5a	--	144.3
6	7.85, dd, (6.8/1.3)	130.4
7	7.95, dd, (8.8,6.9)	133.6
8	8.12, dd, (8.7/1.4)	130.9
9	--	138.5
9a	--	139.1
10	--	--
10a	--	127.4
11	--	166.0
12	3.99, d, (7.2)	28.4
13	5.71, m	120.2
14	--	138.9
15	3.86, s	66.1
	4.68, s (-OH15)	--
16	1.76, s	13.7

1.
